# Attributing climate and weather extremes to Northern Hemisphere sea ice and terrestrial snow: progress, challenges and ways forward

**DOI:** 10.1038/s41612-025-01012-0

**Published:** 2025-05-03

**Authors:** Kunhui Ye, Judah Cohen, Hans W. Chen, Shiyue Zhang, Dehai Luo, Mostafa Essam Hamouda

**Affiliations:** 1 Atmospheric, Oceanic and Planetary Physics, University of Oxford, Oxford, UK; 2 Atmospheric and Environmental Research, Lexington, USA; 3 Department of Civil and Environmental Engineering, Massachusetts Institute of Technology, Cambridge, USA; 4 Department of Space, Earth and Environment, Chalmers University of Technology, Gothenburg, Sweden; 5 Key Laboratory of Meteorological Disaster, Ministry of Education, Collaborative Innovation Center on Forecast and Evaluation of Meteorological Disasters, Joint International Research Laboratory of Climate and Environment Change, Nanjing University of Information Science and Technology, Nanjing, China; 6 Key Laboratory of Earth System Numerical Modeling and Application and Key Laboratory of Regional Climate-Environment for Temperate East Asia, Institute of Atmospheric Physics, Chinese Academy of Sciences, Beijing 100029, China and University of Chinese Academy of Sciences, Beijing, China; 7 Astronomy and Meteorology Department, Faculty of Science, Cairo University, Cairo, Al Giza, Egypt

**Keywords:** Atmospheric dynamics, Climate change, Cryospheric science

## Abstract

Sea ice and snow are crucial components of the cryosphere and the climate system. Both sea ice and spring snow in the Northern Hemisphere (NH) have been decreasing at an alarming rate in a changing climate. Changes in NH sea ice and snow have been linked with a variety of climate and weather extremes including cold spells, heatwaves, droughts and wildfires. Understanding of these linkages will benefit the predictions of climate and weather extremes. However, existing work on this has been largely fragmented and is subject to large uncertainties in physical pathways and methodologies. This has prevented further substantial progress in attributing climate and weather extremes to sea ice and snow change, and will potentially risk the loss of a critical window for effective climate change mitigation. In this review, we synthesize the current progress in attributing climate and weather extremes to sea ice and snow change by evaluating the observed linkages, their physical pathways and uncertainties in these pathways, and suggesting ways forward for future research efforts. By adopting the same framework for both sea ice and snow, we highlight their combined influence and the cryospheric feedback to the climate system. We suggest that future research will benefit from improving observational networks, addressing the causality and complexity of the linkages using multiple lines of evidence, adopting large-ensemble approaches and artificial intelligence, achieving synergy between different methodologies/disciplines, widening the context, and coordinated international collaboration.

## Introduction

Sea ice and seasonal snow (hereafter snow) are two indispensable components of the cryosphere that play an important role in climate variations and change. Their variations modulate surface energy balance and trigger atmospheric circulation response^1–3^. Both Arctic sea ice and spring snow cover in the Northern Hemisphere (NH) have been rapidly decreasing in recent decades amid global climate change^4–6^. Arctic sea ice loss has been considered as a key driver of the Arctic amplification of global warming (AA)^7,8^, amid rapid climate change in the Arctic climate system^9^. Studies have linked changes in sea ice and snow cover with climate and weather extremes manifesting as, for example, heatwaves^10^, droughts^11^ and cold spells^12^, which have caused substantial socioeconomic damages. Understanding the link between sea ice and snow change, and extreme events can potentially enhance predictions and projections of these extremes, contributing to climate change mitigation and adaptation.

Over the past several decades, many studies have attempted to attribute these climate and weather extremes to sea ice and snow change, but the idea remains contentious, and studies are somewhat polarized and fragmented. Drivers of extreme events are multifaceted and attribution of extreme events is challenging^13,14^. Isolating the effects of sea ice and snow change from other competing factors such as internal atmospheric variability and sea surface temperature (SST) may be more complicated than previously perceived. Projected Arctic sea ice loss is not found to significantly drive climate variability and atmospheric circulation change in recent comprehensive climate modeling^15,16^. While autumn snow cover anomalies have been proposed to induce winter extreme weather in the NH^12,17^, their role as a driver of such extremes remains controversial. On the other hand, snow loss, in the form of unusually low snow water equivalent (SWE) usually termed snow droughts^18^, has been increasingly linked with spring-summer extreme events^19^. These have raised questions about whether sea ice or snow change is still a potentially important driver of extreme events and what will be needed to facilitate further significant advances in this area of research.

Climate projections suggest that future extreme events such as heatwaves and extreme precipitation will increase and intensify and that they will tend to break previous records by much larger margins, while Arctic sea ice and snow will continue to decrease according to the Sixth Assessment Report of the United Nations’ Intergovernmental Panel on Climate Change (IPCC AR6)^20,21^. In particular, the earliest Arctic ice-free conditions for September are likely going to occur by 2050 irrespective of emission scenarios^22^. Further, Arctic sea ice extent (SIE) minimum has a downward trend of 12.4 percent per decade from 1979 to 2024 relative to the 1981 to 2010 average with the year of 2012 setting the record minimum (https://nsidc.org/sea-ice-today/analyses/arctic-sea-ice-extent-levels-2024-minimum-set). Spring snow cover extent (SCE) over the NH is projected to decrease by about 8% relative to the 1995–2014 level per degree Celsius of global surface air temperature (GSAT) increase^6^. Both the years 2023^23^ and 2024 successively broke the temperature records with the latter at about 1.55 °C above pre-industrial level according to the World Meteorological Organization (https://wmo.int/news/media-centre/wmo-confirms-2024-warmest-year-record-about-155degc-above-pre-industrial-level). These suggest global climate change may evolve beyond our current understanding and projections. There is a window of opportunity to accelerate progress in attributing climate and weather extremes to sea ice and snow change so that critical information can be used to assist the predictions of these extremes and to mitigate the societal and ecological damages linked with a changing cryosphere. Therefore, there is an urgent need to review our current progress in attributing climate and weather extremes to sea ice and snow change and to suggest a way forward in this area of research.

Previous review papers have focused on the impacts of sea ice or snow on climate and weather variability^2,24,25^. In this review, we synthesize the major progress in attributing climate and weather extremes to sea ice and snow change in the NH in terms of observed linkages, physical pathways and uncertainties. While sea ice and snow are often considered separately in many studies for attributing extreme events, there has been an increasing trend to consider their combined influences^26,27,28^. We consider both sea ice and snow in this review, both of which are highly sensitive and vulnerable to global warming and climate change, to highlight some of their shared mechanisms and their combined influences on extreme events. This approach emphasises the benefits of considering the influence of both sea ice and snow changes on extreme events under the same framework and highlights the cryospheric feedback to the climate system. Built on the recent advances and emerging opportunities, we suggest ways forward to make further advances in this area of research. This review will thus serve as a bridge between current progress and future advances in attributing climate and weather extremes to sea ice and snow change.

## Observed linkages between a changing cryosphere and extremes

**Fig. 1:**
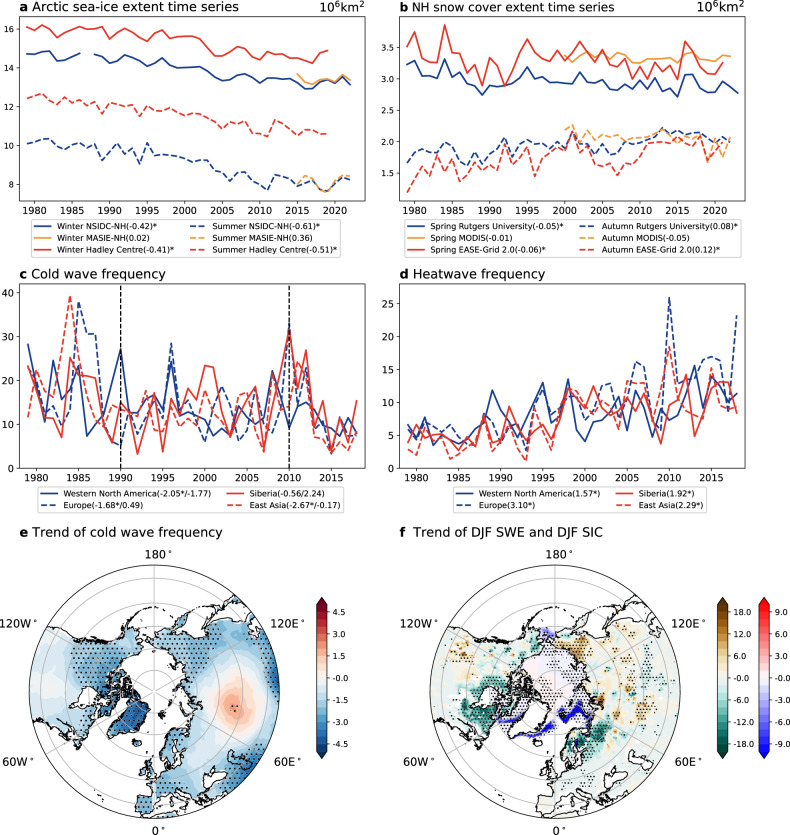
Temporal evolution and trends in Arctic sea ice, snow, cold waves, and heatwaves in the Northern Hemisphere. **a** Arctic sea ice extent in winter (solid line) and summer (dotted line) from the National Snow and Ice Data Center (NSIDC) (blue lines; Fetterer et al.^256^; Temporal coverage: 26 October 1978 to present, spatial resolution: 25 km × 25 km), the Multisensor Analyzed Sea Ice Extent - Northern Hemisphere (yellow lines; Fetterer et al.^257^; Temporal coverage: 1 January 2006 to present, spatial resolution: 1 km x 1 km), and Met Office Hadley Centre observations datasets (red lines; HadISST.2.2.0.0, Titchner and Rayner^258^; Temporal coverage: 16 January 1850 to present, spatial resolution: 1°×1°). **b** Northern Hemisphere snow cover in spring (solid line) and autumn (dotted line) from Rutgers University Global Snow Lab (blue lines; Robinson et al.^259^); Temporal coverage: 1 January 1979 to present), the Moderate Resolution Imaging Spectroradiometer (MODIS) (yellow lines; Hall et al.^260^; Temporal coverage: 1 March 2000 to present, spatial resolution: 0.05° × 0.05°), and NSIDC EASE-Grid 2.0 (red lines; Brodzik et al.^261^; Temporal coverage: 23 October 1978 to 1 January 2023, spatial resolution: 25 km × 25 km). Decadal trends are provided in the legends in both **a** and **b**. **c** Cold wave frequency defined by the Excess Cold Factor and **d** Heatwave frequency defined by the Excess Heat Factor over Western North America (blue solid line, 30°N-50°N, 130-110°W), Europe (blue dotted line, 35°N-70°N, 15° W-30°E), Siberia (red solid line, 60°N-70°N, 90-140°E), and East Asia (red dotted line, 30° N-45°N, 100-135°E) from Gridded land surface extremes indices (HadEX3; Dunn et al.^262–264^). The two black dotted lines represent the years 1990 and 2010, respectively. The first (second) numbers in the legends indicate the decadal trends in the entire period (1990-2010). The * in the legends represents trend values statistically significant at the 5% level. **e** Spatial distributions of decadal trends in cold wave frequency. **f** Spatial distributions of decadal trends in winter snow water equivalent (shading; yellow-green colorbar) from the ESA DUE GlobSnow project funded by the European Space Agency (v3.0, bias-corrected monthly data). (Temporal coverage: 1 January 1979 to 31 December 2018, spatial resolution: 0.25° × 0.25°) and winter sea ice concentration (shading; red-blue colarbar) from Met Office Hadley Centre. Dotted areas in e and f are statistically significant at the 5% level. All trends are calculated using Theil-Sen estimate of linear trend and Mann-Kendall trend significance; Kendall^265^. All data have been linearly interpolated onto a 1° × 1° latitude-longitude grid. Units of decadal trends: 10^6^ km^2^ for sea ice extent and snow cover extent, % for sea ice concentration, days for cold wave and heatwave frequency, and mm for Snow water equivalent.

Both SIE and sea ice concentration (SIC) in the Arctic have been decreasing during the satellite era (Fig. 1a, f), consistent with the global warming trend in GSAT. The Arctic has also lost more than 2 × 10^6^ km^2^ of multiyear sea ice over the scatterometer record (1999–2017) (Kwok, 2018). An ice-free Arctic, defined as having less than 1 million square kilometers of September sea ice area, is projected to occur by 2050 independent of emission scenarios^22^. There are also concurrent downward trends in NH spring SCE (solid lines, Fig. 1b), and in winter snow water equivalent (SWE)^29^ in North America and west Eurasia (Fig. 1f). Both the summer sea ice decline and spring SCE decline are considered to be of very high confidence according to IPCC AR6^20^. The autumn SCE in the NH shows contrastingly significant upward trends (blue and red dashed lines, Fig. 1b), though the recent decades of Moderate Resolution Imaging Spectroradiometer data^30^ show weakly insignificant downward trends (yellow dashed line). Trends in the NH autumn snow cover are less monotonic and projections of them are less confident than those for spring^31^. For the lowest five NH SCE values for each month in the period of March–June, seventeen out of twenty values occurred after 1990 between 1967 and 2015^32^. Snow drought durations over eastern Russia, Europe, and western United States have lengthened by ∼2, 16, and 28%, respectively, in the latter half of 1980 to 2018^18^. Causes of the changes in Arctic sea ice are found to be related to the Arctic dipole^33^, Arctic cyclones^34^, sea ice thinning^35^ or a combination of different factors^36^. An extreme SIE minimum of the magnitude seen in 2012 is not likely to occur without anthropogenic influence^37^. Cold season Arctic cyclone number has a strong positive trend from around 2000 with a causal connection to both the warm and cold season Arctic sea ice loss, which suggest that cyclones caused changes in the sea ice^38^. August cyclones have become more destructive in accelerating Arctic sea ice loss during 2009–18 compared to 1991–2000^39^. Moreover, Arctic minimum SIE is jointly influenced by the strength of the cyclone, its timing and its location relative to the sea ice edge^40^. In addition, multidecadal variability in Arctic sea ice has been reported^41–45^, though it is less clear for snow. The extent to which recent trends in Arctic sea ice and snow are influenced by multidecadal variability remains to be clarified.

Concurrently, hot extremes have increased and extreme precipitation has intensified while cold extremes have decreased according to IPCC AR6 (see also Fig. 1c, d). The increase in heat extremes is evident while the trend in cold extremes is mixed to some extent with significant decreasing trends over many regions but no significant change in central Eurasia (Fig. 1c-e). The decreasing (increasing) trends in cold (heat) extremes are mostly consistent with global warming. Counter-intuitively, cold extremes have increased over some regions in the NH over the recent decades^46^ (see also Fig. 1c, e). A recent study found that deaths from cold temperatures have increased over the period of Arctic amplification^47^. Furthermore, persistent severe winter weather in the United State including extreme cold and heavy snowfall increased the interest in the connection between extreme winter weather and Arctic change including Arctic sea ice and snow cover (https://www.theguardian.com/us-news/2025/jan/22/cold-weather-storm-emergency-south).

**Fig. 2:**
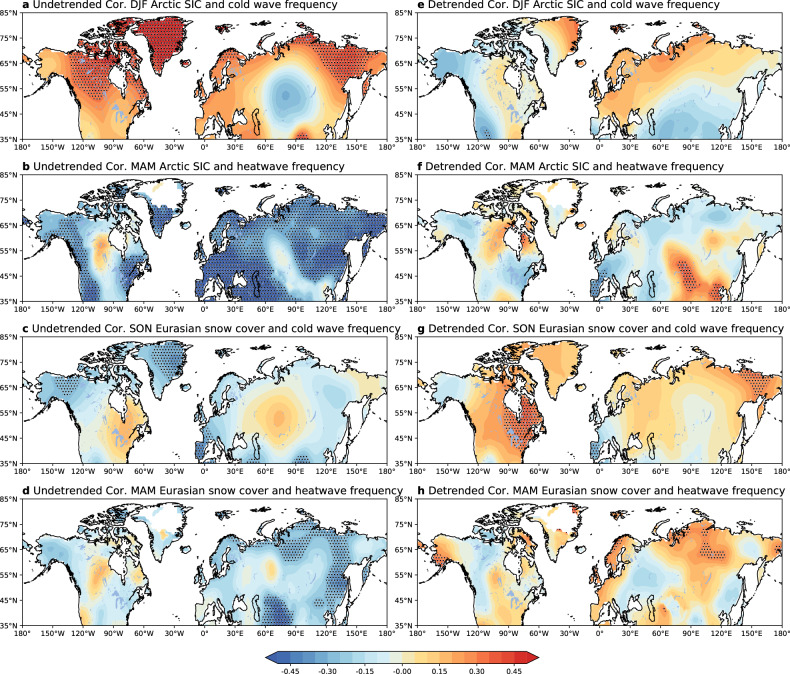
Relationships between Arctic sea ice (HadISST.2.2.0.0), Eurasian snow (NSIDC) and cold/hot extremes (HadEX3) for the period 1979–2018. Pearson correlation coefficients (**a**) between winter Arctic sea ice concentration (north of 60°N) and cold wave frequency, (**b**) between spring Arctic sea ice concentration index (north of 60°N) and heatwave frequency, (**c**) between autumn Eurasian snow cover (35-70°N, 40-120°E) and cold wave frequency, and (**d**) between spring Eurasian snow cover (35-70°N, 40-120°E) and heatwave frequency. Dotted areas are statistically significant at the 5% level. **e**–**h** are the same as in **a**–**d**, but for detrended Arctic sea ice concentration, Eurasian snow cover, and heatwave/cold wave frequency by subtracting trends estimated by Theil-Sen estimate of linear trend from the data.

Owing to observed concurrent trends in both the extremes and the sea ice/snow, there has been surging interest in studying sea ice/snow as potential drivers of historical climate and weather extremes^48^. There are some statistically significant correlations between sea ice, snow cover and cold/hot extremes in the NH (Fig. 2a–d, left panels). This suggests potential physical linkages between them and studying their relationships will benefit predictions of extreme events. After removing the trends in the data, the correlation patterns are weaker, change sign or are strengthened depending on region (Fig. 2, right panels). The detrended correlation patterns suggest that more autumn snow cover is linked with increasing cold wave frequency over majority of the NH (Fig. 2g) while less sea ice and snow decrease is linked with increasing heatwave frequency in some regions, albeit not statistically significant, but with statistically significant reduced heatwave frequency in Europe, Russia and east Asia (Fig. 2f, h). The detrended correlation patterns also show that Arctic sea ice loss is however linked with cold wave frequency decrease over a large part of the NH but also with cold wave frequency increase in some regions for example, western North America and Asia (Fig. 2e). One plausible explanation for the change in correlation strength/sign is that the relationships between sea ice, snow cover and cold/hot extremes include linear/nonlinear coupling and impacts of timescale-dependence processes, for example the long-term climatic trends. These relationships are complicated given the multiple climate feedback processes in the climate system under global warming. Attribution of climate and weather extremes to sea ice and snow may be far more challenging than our current understanding indicates. In particular, Arctic sea ice loss has been a controversial driver of the recent counter-intuitive increasing cold extremes in some regions of the NH^46,49,50^. Notwithstanding the debate on whether sea ice and snow change is physically linked with cold extremes, more attention than would otherwise be expected has been drawn to the changing cryosphere and its climatic feedback in global climate change. Existing efforts by the research communities suggest that the changing cryosphere’s role in driving climate and weather extremes should not be neglected and has the potential to improve predictions of these extremes. Improving their prediction from the perspective of cryospheric influence is clearly beneficial^51^.

A number of extreme events, of various types, seasons and regions, have been linked with the change in sea ice and snow. We summarize the most frequently seen types of extremes in the literature that are attributed to sea ice and snow change (see also Table 1).

**Table 1:** Summary of extreme events linked with sea ice and snow change the Northern Hemisphere in selected relevant studies

Reanalysis/observations-based studies are in plain text. Studies that include modeling are highlighted in bold. Modeling is counted if numerical models are run or output from these models are analyzed.

### Cold extremes

The most known linkage in terms of scientific interest and societal impact and most focused on in existing studies is less Arctic sea ice and more Eurasian snow contributing to winter cold extremes in the NH. These cold extremes usually cause large-scale societal impacts and represent a challenge for climate and weather forecasting. More specifically, more snow cover in early autumn, particularly in October and November, and winter over Eurasia is argued to drive winter cold extremes in North America and Eurasia^12,52^. This relationship is reported in a number of studies using observations and climate modeling^53–56^. On the other hand, there have been relatively less clear relationships between snow cover in North America and extreme events, though there have been some studies on the impacts of snow change on atmospheric circulation^57–60^. Arctic sea ice loss in autumn and winter is also considered to induce cold extremes in North America and Eurasia^61^^,62^. This linkage has been far more studied and debated than the snow counterpart in recent years. There are however polarized views on this linkage with some studies supporting^62,63^ and other studies questioning its robustness^50,64–66^. Arctic sea ice loss in the summer of 2011 is thought to contribute to the extreme cold event in the Asian continent during late January–early February 2012^67^. A recent study has indicated that winter sea ice loss in the Kara Sea could potentially drive cold surges over the tropical Western Pacific^68^. Cold extremes are expected to increase moderately in response to projected Arctic sea ice loss, over Eurasia, particularly in the Asian region^15,16^.

### Heatwaves

Heatwaves in spring and summer across the NH including the United States, Europe, Russia, the Mediterranean and Asia have also been linked with sea ice and snow change. For these linkages, both trends in heatwaves and some specific heatwaves are discussed in existing studies. For example, the Arctic sea ice loss in summer may have played a crucial role in both the 2010 and 2016 Russian summer heatwaves^69^. Extreme heatwaves in June 2021 over Europe are also largely attributed to high latitude Eurasian snow loss in April and May^70^. The upward trends in European summer heatwaves since the 1970s are suggested to be driven by the decline in Arctic sea ice and Eurasian snow^27^. The leading mode of interannual variation of spring extreme heat events over mid-to-high latitude Eurasia in the recent two decades is also dominated by the winter Arctic sea ice anomaly^71^. Arctic sea ice loss is particularly deemed a driver of heatwaves over various regions in China^72,73^, and may modulate the spatial distribution of North China heatwaves^74^. Arctic sea ice loss is also considered as a strong modulator of the summertime heatwaves over the United States/western North America in recent decades^10,75^. Sea ice loss may be a strong modulator of the extreme (1000-year weather event) heatwave^75^ in June and July 2021 over the Western United States^76^, causing devastating impacts over Western North America. Snow cover in the Tibetan Plateau (TP) has been linked to summer heatwaves in Eurasia^77–80^. Snow droughts, exhibiting unusually low snow mass, in late winter has been suggested as a key driver of early spring heatwaves in the same region over the NH, particularly over Eurasia^19^. Further, the snow drought in April/May 2021 over the Western United States^81^ was followed by the extreme heatwave in June and July 2021 over Western North America.

### Extreme precipitation and extreme snowfall

Extreme precipitation and extreme snowfall are also attributed to Arctic sea ice and snow change. October snow cover in central Siberia is linked with subsequent spring extreme precipitation frequency in southern China^82^. Spring snow cover over Eurasia is found to be closely linked to extreme precipitation over southwestern Xinjiang during 2003–2018^83^. Spring snow cover over the TP is also considered to have driven the extreme precipitation events in Pakistan in July and August 2022^84^. Arctic sea ice loss has been linked to an increase in snowfall in the cold seasons^55,85^. Specifically, the extreme European snowfall in February 2018 is considered to be mostly caused by the Arctic sea ice loss^86^. Arctic sea ice loss is linked to winter extreme precipitation over the TP^87^, and has also been shown to increase extreme precipitation events over the Mediterranean region^88^. The sea ice loss in May over the Kara Sea is found to play an important role in driving the extreme July Meiyu-Baiu rainfall in 2020^89^. Arctic sea ice loss in late spring-early summer has at least partially contributed to the extreme June-July Meiyu-Baiu rainfall in 2020^90,91^, and sea ice loss in July has contributed to the record-breaking July-mean precipitation in North China in 2021^92^. Arctic sea ice loss in summer together with SST anomalies in the north-western Arabian Sea is also suggested to cause more frequent late season Indian Summer Monsoon Rainfall extremes^93^.

### Wildfires

Wildfires in tundra have major impacts on regional and global carbon and energy dynamics as they impact the carbon stock in the soil and permafrost^94^. Both Arctic sea ice loss and earlier snowmelt have been increasingly linked with wildfire activities in a few studies and further evidence for such linkage is still emerging. Arctic sea ice loss has long been suggested to contribute to wildfires in the NH^95,96^. Recent studies have linked sea ice loss in the NH to wildfires in eastern Siberia^97^ and in the Southern Hemisphere to wildfires in Australia^98^. Earlier snowmelt is found to cause earlier ignitions in North America and may increase future fires in the west of North America^99^. Earlier snowmelt is also considered to contribute to recent extreme Siberian fire seasons^100^, and earlier fire ignitions in North America between 2001 and 2019^101^. Snow reduction in the form of snow cover reduction and less snowmelt has also been linked with spring wildfire burned area in West Siberia^102^.

### Droughts

Snow is an important form of water storage during cold months and the subsequent melt thus provides vital water resources for agricultural usage and drinking. Snow loss and earlier snowmelt contribute to agricultural/meteorological droughts by impacting availability and timing of melt water^11^^,^^81^^,^^103^^,^^104^^,^^105^. Research on this linkage is still limited and mainly focuses on regional scales due to data availability and complicated processes involved. For sea ice, early‐autumn sea ice in the East Siberian Sea has been linked with drought conditions in June in Northwest China^106^.

### Extreme haze pollution

Extreme haze events in China are considered to be driven by Arctic sea ice loss in autumn and early winter^107^^,^^108^. These extreme haze events are associated with wintertime air stagnation and continuing Arctic sea ice loss may make them occur more frequently.

### Compound extremes

There has been limited research on compound extremes that have been attributed to sea ice and snow change. Snow droughts are frequently followed by extreme heatwaves, hydrological and agricultural droughts^11,19,104^. These are categorized as compound extremes. Spring TP snow cover has been suggested to more strongly drive summer compound heatwaves in Western Europe after 1998 compared to the period before^109^. The variability of extreme summer multivariate compound heatwaves over Western Europe for the period 1979 to 2021 has been largely attributed to the combined effects of North Atlantic SST, TP snow cover, and Arctic sea ice^110^.

## Major mechanisms and pathways

**Fig. 3:**
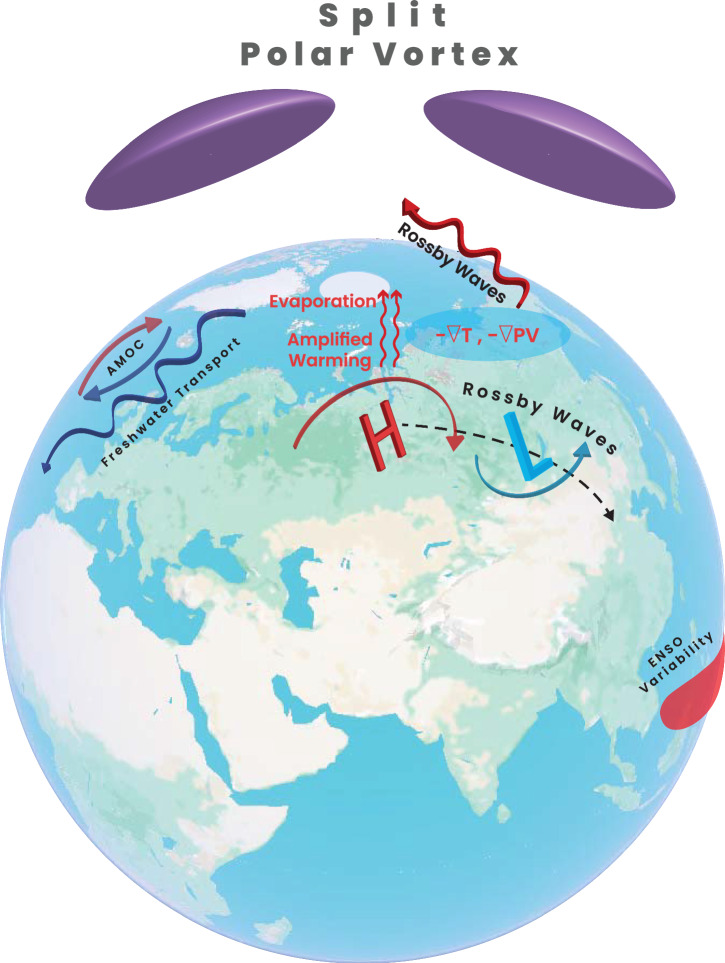
Major pathways for sea ice to impact atmospheric circulation and weather/climate variability. Sea ice loss induces warming to weaken the meridional temperature gradient (-**∇T**), often hypothesized to lead to a meandering jet stream, and also to weaken the potential vorticity (**-**∇**PV**) gradient, which is thought to favor atmospheric blocking. Warming induced by sea ice loss modulates vertically propagating Rossby waves to the stratosphere (upward wavy thick arrows), causing the stratospheric polar vortex, for example, to split. Directly excited Rossby wave trains by sea ice loss (curved arrows denoted by ‘H’ and ‘L’.) can propagate to lower latitudes and the Pacific Ocean. Enhanced evaporation from sea ice loss (red thin wavy arrows) is linked to extreme snowfall and extreme precipitation. Arctic warming and sea ice melt weaken the AMOC by increasing freshwater input to the North Atlantic (blue wavy arrows). A weakened/collapsed AMOC results in cooler winters in northern and western Europe. Arctic sea ice loss is also linked to variabiliyt of El Niño–Southern Oscillation.

**Fig. 4:**
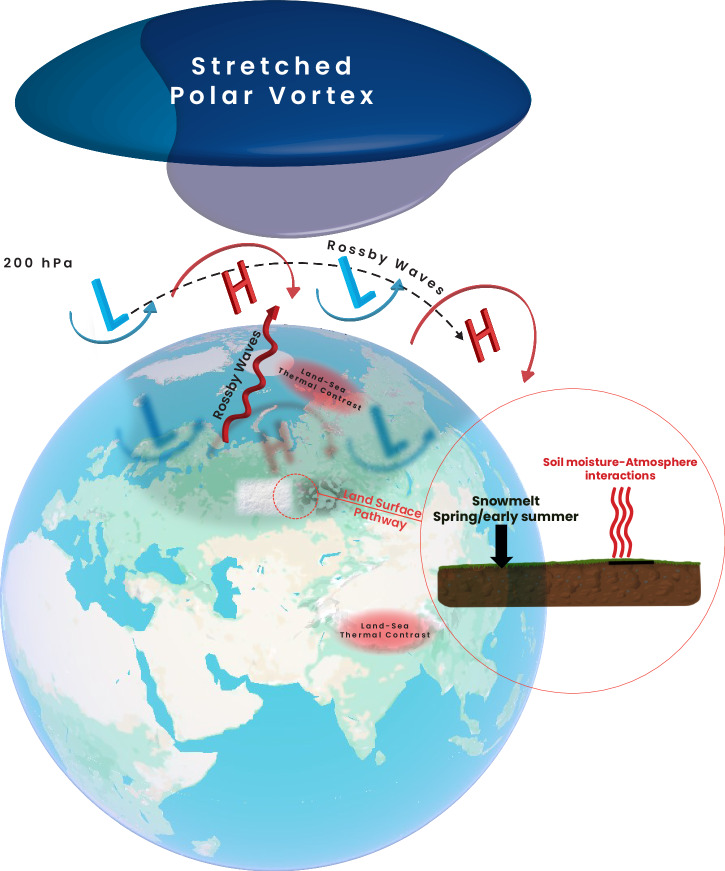
Major pathways for seasonal snow to impact atmospheric circulation and weather/climate variability. A stronger Siberian High is usually observed in response to an increase in autumn snow cover, which is accompanied by anomalous vertical wave activity to the stratosphere, (upward thick red wavy arrows) causing polar vortex disruptions, for example by inducing stretching of the stratospheric polar vortex. The land-sea thermal contrast is usually enhanced by earlier snowmelt or less snowmelt, causing, for example, stronger Asian monsoon flow. The land-surface pathway involves snowmelt in spring/early summer, which affects surface albedo, surface runoff and soil moisture, and induces subsequent soil moisture–atmosphere interactions. This effect strongly depends on soil moisture memory, which is still an active topic of research.

Sea ice and snow are usually treated as boundary conditions for driving climate and weather variability. Both sea ice and snow have a high reflectivity of shortwave radiation and act as an efficient insulator of sea surface and land surface, respectively. These two features relate to the albedo effect and allow sea ice and snow to strongly modulate surface energy balance by altering net incoming shortwave radiation. Sea ice and snow loss thus causes surface warming and vice versa, which induces surface heating anomalies. The albedo effect is expected to be minimal at high latitudes during winter months. There are however two important non-albedo effects associated with sea ice and snow, respectively. For sea ice, fractional anomaly affects turbulent heat fluxes from the relatively warm ocean surface to the atmosphere in cold months, causing surface heating anomalies. Melting of sea ice also provides freshwater input to the ocean and affects ocean salinity and circulation. For snow, melting affects the soil moisture during the snowmelt season (usually spring-early summer) and subsequently land-atmosphere interactions, referred to as snow hydrological effect. Melting of snow on ice sheets also modifies albedo, changing the underlying surface ice melt and the overall ice sheets dynamics. The resulting fresh water input from snow melting to the oceans can impact on salinity and ocean dynamics. In addition, snow cover is a driver of the permafrost thermal state change owing to its insulating effect^111^^,^^112^^,^^113^. Snow melt in spring can also influence the onset of seasonal ground warming and permafrost thaw^111^. Snow thus modulates the response of permafrost under a warming climate, which has far-reaching effects on climate change and infrastructure at high latitudes. A range of pathways and mechanisms have been proposed to explain the role of sea ice and snow change in driving atmospheric circulation changes, and impacting climate and weather variability including extreme events. These are predominantly atmospheric and land surface pathways but the oceanic pathway is emerging. We here summarize major pathways and mechanisms that have been proposed and recognized widely in the literature in terms of influence of sea ice (see also Fig. 3) and snow change (see also Fig. 4).

### Equator-to-pole temperature gradient

Amplified Arctic warming – strongly contributed to by sea ice loss – is nearly four times as large as the global average^114^. Arctic sea ice loss thus weakens the equator-to-pole temperature gradient, which has far-reaching effects on the atmospheric circulation. One such effect suggested is a wavier midlatitude jet and slower-moving weather systems^115^^,^^116^. These favor cold air intrusions from the Arctic and are linked to recent cold extremes in the NH^116^. However, there have been polarized views on this effect with the topic arguably being the most contentious with regard to the impact of Arctic sea ice loss and AA on midlatitude weather^117^^,^^118^^,^^119^^,^^120^. The effect is sensitive to metrics used to characterize sinuosity of the atmospheric circulation^117^^,^^118^. Recent comprehensive climate modeling suggests that the midlatitude jet shifts equatorward and weakens in response to projected Arctic sea ice loss, although the effect is relatively small^15^^,^^16^^,^^121^. However, aquaplanet simulations show that weakened jet streams do not necessarily become wavier^122^. Two other effects frequently mentioned in the literature associated to warming anomaly induced by Arctic sea ice loss are changes in atmospheric blocking and storm track^123^^,^^124^. Increases in atmospheric blocking frequency and equatorward shifts of the storm track usually accompany weakened westerlies and equatorward shifts of the midlatitude jet in response to Arctic sea ice loss^15,16^. Arctic warming related to Arctic sea ice loss has been shown to enhance atmospheric blocking via positive feedbacks^123^. However, the persistence and quasi-stationarity of atmospheric blocking more strongly depends on the meridional potential vorticity (PV) gradient than the meridional temperature gradient^123^^,^^125^.

### Land-sea thermal contrast

There has been little mention of equator-to-pole temperature gradient change for snow change. Snow change-induced surface warming/cooling is mostly linked to land-sea thermal contrast particularly in spring and summer. For example, earlier snowmelt in northeastern Siberia is suggested to warm the land surface to enhance the thermal contrast between the land surface and the Arctic Ocean and therefore facilitates the formation of the Arctic front jet in early summer, causing anomalously high lightning and fire activity^100^. The land-sea thermal contrast between the Eurasian continent and the surrounding oceans is also a major mechanism for Eurasian snow impacting summer monsoons in Asia^126^^,^^127^^,^^128^. There are a variety of atmospheric circulation changes associated with snow change in Eurasia including western Pacific subtropical high, subtropical jets, upper tropospheric easterlies, and trade winds in the eastern equatorial Pacific Ocean^126^^,^^129^.

### Rossby wave excitation

Direct Rossby wave excitation – due to surface heating anomaly from Arctic sea ice loss and snow change – is proposed in some studies. The excited Rossby waves usually propagate eastward or southeastward to influence climate and weather over the Eurasian region and further downstream^48^^,^^130^^,^^131^. However, the direct response to sea ice loss often manifests as a heat low and favorable relative vorticity gradient is needed to trigger Rossby wave response but the response is relatively weak^16^^,^^132^. This implies that direct Rossby wave excitation from Arctic sea ice loss is likely weak and highly circumstantial^132^^,^^133^. There are suggestions that Arctic sea ice impacts the background state to modulate the propagation of midlatitude Rossby waves which impacts the lifetime or persistence, movement and intensity of atmospheric blocking^123^^,^^125^^,^^133^. There are studies also suggesting indirect forcing and amplification of Rossby waves in mid-high latitudes by sea ice loss through forcing anticyclonic anomaly via transient eddy vorticity fluxes^108^. For snow change, excessive Eurasian snow cover is shown to lead to Rossby wave circulation response and weaken the East Asia summer monsoon^130^^,^^134^. Snow cover anomalies in the TP are also suggested to indirectly excite Rossby waves via influencing SSTs over the Atlantic and Pacific Oceans, which strongly drove the 2003 European summer heatwave^80^. Snow cover anomalies in the TP are also linked to albedo change that excites Rossby waves to impact downstream regions^135^. Low spring North American snow cover also excites a direct stationary Rossby wave response to induce an anomalous anticyclone over Greenland, contributing to wavier summer atmospheric circulation over the North Atlantic^136^.

### Meridional potential vorticity gradient

**Fig. 5:**
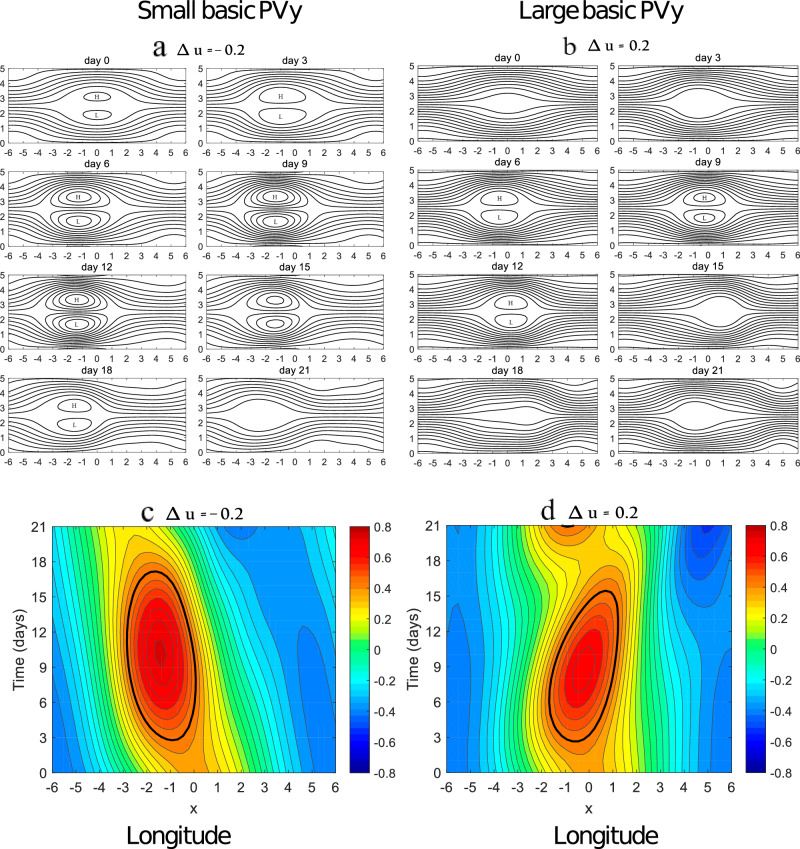
Smaller basic meridional potential vorticity increases atmospheric blocking lifetime and zonal extent. **a**, **b** Planetary-scale fields of idealized blocking flows under small and large basic meridional potential vorticity gradient conditions based on a baroclinic nonlinear multi-scale interaction model. **c**, **d** The corresponding Time-Longitude evolution of the blocking amplitude. This figure is adapted from Luo and Zhang (2020), permitted under the American Meteorological Society Copyright Policy. Journal of the Atmospheric Sciences 77, 7; 10.1175/JAS-D-20-0004.1.

Similar to the equator-pole temperature pathway, the PV gradient is another emerging pathway for Arctic sea ice loss to influence midlatitude weather. As noted above, Arctic sea ice loss does not significantly excite Rossby waves^133^. Instead, it can reduce the meridional background temperature gradient (Ty) and meridional basic PV gradient (PVy) via enhanced Arctic warming. Nevertheless, the magnitude of PVy is a more important factor for the evolution of atmospheric blocking than Ty^137^. In fact, intense weather extremes in mid-high latitudes are often closely linked to increased persistence and stationarity of atmospheric blocking events (10–20 days)^138^. The temporal evolution of atmospheric blocking is directly tied to PVy rather than Ty according to the nonlinear multi-scale interaction (NMI) model of atmospheric blocking^125^^,^^139^^,^^140^^,^^141^, even though PVy includes Ty^142^. In this NMI model, atmospheric blocking is regarded as a Rossby wave packet described by a nonlinear Schrödinger equation forced by synoptic-scale eddies^142^. The energy dispersion of the blocking system is proportional to PVy, whereas its nonlinearity is inversely proportional to PVy (i.e., 1/PVy)^139^. When PVy is smaller, the blocking system has weaker dispersion and stronger nonlinearity so that atmospheric blocking can have longer lifetime, larger zonal scale, slower decay and weaker eastward movement^140^^,^^142^ (Fig. 5), favoring intense cold extremes.

However, Arctic warming is not necessary for midlatitude weather extremes because it is not the only factor leading to the PVy reduction^125^, thus explaining the intermittency and uncertainty of the Arctic-midlatitude linkage^143^. In the NMI model, there is a critical threshold of PVy that determines the persistence of atmospheric blocking^139^. When PVy is below this critical threshold under much stronger winter Arctic warming, atmospheric blocking becomes less persistent in a certain region and shows stronger westward movement^144^, therefore suppressing midlatitude cold extremes^138^. This explains why winter cold extremes have undergone a sharp decline under high-emission scenarios^145–147^.

### Evaporation from the Arctic ocean

Sea ice loss exposes underlying ocean surface and is thus suggested to increase local ocean evaporation and atmospheric moisture content^148–150^. This has been linked to local enhanced precipitation^148^ and extreme snowfall in Europe^86^. Evaporation in the Arctic had increased between 2003 and 2013^150^. Enhanced evaporation from the Arctic marginal seas has been linked with increased land precipitation, with a 16% increase for per million square kilometers loss in sea ice area during 1980–2021^151^. Arctic sea ice loss for the end of the twenty-first century has been shown to be a major contributing factor for local enhanced precipitation and freshwater input, signaling an amplified Arctic hydrological cycle^148^.

### Stratospheric pathway

The stratospheric pathway is mostly active in cold months involving troposphere–stratosphere coupling when the Northern stratospheric polar vortex (NSPV) is strong and active. Regarding the locations of sea ice or snow change for modulating the stratospheric pathway, the Barents–Kara Seas (BKS)^63,152^ and the Siberian region^12,17,31,53^ are frequently cited as key regions. The BKS region exhibits the largest sea ice loss and surface warming over the Arctic in fall and winter, which would represent substantial heating forcing. BKS warming is also closely linked with Ural atmospheric blocking activity, and high-pressure anomalies in the Ural region strongly modulate vertical planetary wave forcing to impact the NSPV^153^. The Siberian region has the maximum of upward wave activity flux during winter months in terms of climatology^154^, has a strong topographic forcing of planetary waves and has one of the most intense semi-permanent high pressure systems – the Siberian High. These features may explain why sea ice change in the BKS and snow change in the Siberian region have a stronger modulating effect on the vertical planetary wave propagation. Perturbations to the NSPV by sea ice and snow change due to modulation of vertical propagation of planetary Rossby waves have been suggested as a major pathway. The NSPV is usually weakened by enhanced vertical planetary Rossby waves linked with sea ice loss and snow increase^12,31,155,156^, the latter of which is usually accompanied by a stronger Siberian High^12^. Sudden stratospheric warming is usually observed to accompany the disruption of the NSPV. However, NSPV stretching events, which is a less well-known type of NSPV disruptions, is followed by more-extreme cold spells in North American^56^. Snow anomalies tend to better induce NSPV stretching events than sea ice^56^. However, constructive linear interference between the anomalous wave and the climatological wave is proposed to be a necessary condition for stratospheric links to be effective^157^. The subsequent downward influence of the disrupted NSPV has been linked to cold extremes and extreme winter haze in the NH^12^^,^^56^^,^^107^. Related to this delayed downward influence, the stratospheric pathway provides potential for improving subseasonal to seasonal forecasts and is thus of substantial scientific interest.

### Land surface pathway

The land surface pathway is mainly discussed in terms of the impacts of snow change on albedo, soil moisture and subsequent land-atmosphere interactions. However, there are also discussions on the memory effects of snow and soil temperature on bridging summer sea ice loss to following winter temperature anomalies in Eurasia^158^ and the effect of snow cover on soil temperature^159^. Snowmelt and snow fraction affect surface temperature via albedo effects^3,160^. Snowmelt supplies important freshwater to land surface, affects soil moisture content and impacts climate and weather via the hydrological effect^129,161–165^. This involves soil moisture-atmosphere interactions and land surface-atmosphere interactions^166^. Earlier snowmelt and less snowmelt due to low snow mass therefore lead to warmer and drier land surfaces^167^ which diminishes the land’s capacity to cool itself through evaporation and thereby increases upward sensible heat fluxes. A warmer and drier land surface is therefore conducive to heatwaves, wildfire activities, and compound droughts/heatwaves^19,102,168,169^.

**Fig. 6:**
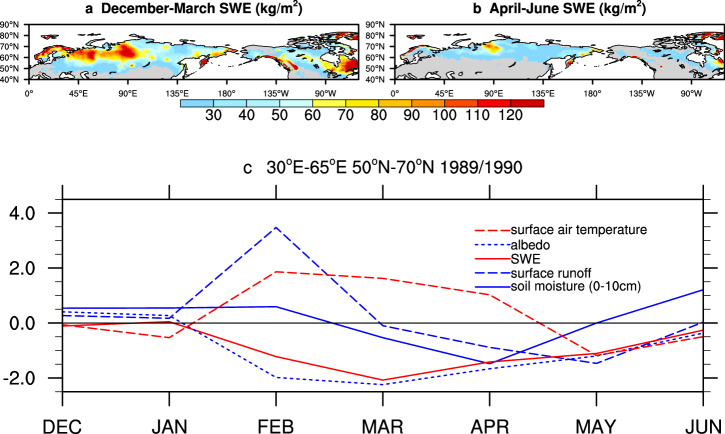
An example of the major processes in snow mass anomaly impacting land surface and climate variability. **a**, **b** Spatial distribution of SWE ( > = 5 kg/m^2^) for December-March and April-June averages. **c** Standardized anomaly of area-mean of various variables for 1989/1990 against the 1980–2010 period. The domain for computing the area-mean is indicated in panel **c**. Data is from NOAH land surface model output from version 2 of the Global Land Data Assimilation System (GLDAS) designed by the National Aeronautics and Space Administration of USA. GLDAS ingests satellite- and ground-based observational data products, using advanced land surface modeling and data assimilation techniques, in order to generate optimal fields of land surface states and fluxes.

An example of this land surface pathway is given in Fig. 6 using NOAH land surface model output from version 2 of the Global Land Data Assimilation System^170^. Snow accumulates during cold months (Fig. 6a) and snowmelt usually occurs during April-June in the NH (Fig. 6b). Rapid snowmelt and low snow mass (solid red, Fig. 6c) in February-May of 1990 led to a surge in initial surface runoff (long-dashed blue line), a persistent decrease in surface albedo (short-dashed blue line) and higher surface temperature, as well as less surface soil moisture in March and April.

### Oceanic pathway

The oceanic pathway has been relatively less explored but results of the influence of sea ice loss on major climate modes are emerging. Locally, Arctic sea ice loss has been found to modulate freshwater content of the Beaufort Gyre^171,172^. On seasonal to interannual timescales, Arctic sea ice loss is linked with El Niño-Southern Oscillation via ocean-atmosphere interactions in observational analysis and climate modeling^173–175^. Arctic warming and sea ice melt can weaken the Atlantic Meridional Overturning Circulation (AMOC) by increasing freshwater input to the North Atlantic^176^. This influx of freshwater reduces the water salinity and density in key regions of deep water formation. As a result, the sinking of surface waters in descending branches of the AMOC is weakened. A weakened AMOC would lead to an overall cooling over most of the NH under the background of global warming^177^, while a complete collapse of the AMOC would lead to substantially cooler winters in northern and western Europe with temperature decreases of up to 5° to 15 °C within a century^178^. It has also been suggested that a slowdown of the AMOC can increase the risk of severe weather by increasing baroclinicity^179^. Some studies have estimated an AMOC collapse around the mid-century^180^. However, there are also studies that argue that the recent AMOC slowdown is within the range of internal multidecadal variability^181^, and that mechanisms such as strengthened overturning circulation in the Nordic Seas can act as a stabilizing factor for the AMOC^182^. While most CMIP6 climate models project a slowdown of the AMOC in the later twenty-first century, it does not necessarily mean a significantly weakened Gulf Stream, as the latter is also partially driven by the North Atlantic Subtropical Gyre^183^.

Results on the oceanic pathway for snow anomalies to impact climate and weather variability are still limited but there have been suggestions that this pathway may be important in linking snow anomalies to extreme events^60,80^.

### Combined influence

The combined influence of sea ice and snow loss has been proposed and studied in some studies for autumn-winter and spring-summer seasons. Instead of considering individual effects of sea ice and snow change, their combined effects therefore have increasingly attracted research interests. For autumn-winter seasons, the major pathway for combined influence is the troposphere–stratosphere coupling^26^^,^^107^^,^^184^. The major mechanisms for spring–summer seasons include weakening equator-pole temperature gradient to reduce mid-high latitude zonal winds and shift the jet stream^184^, and exciting Rossby waves^27^. Recent understanding of the combined influence of sea ice and snow change is tied to the three poles warming, namely over the Arctic, the Antarctica and the TP, particularly contributed to by sea ice/snow melting^28^ (see also Fig, 7). This has driven hemispheric-scale atmospheric circulation change including the poleward movement of westerly jet streams, amplified Rossby waves and expansion of subtropical highs.

**Fig. 7:**
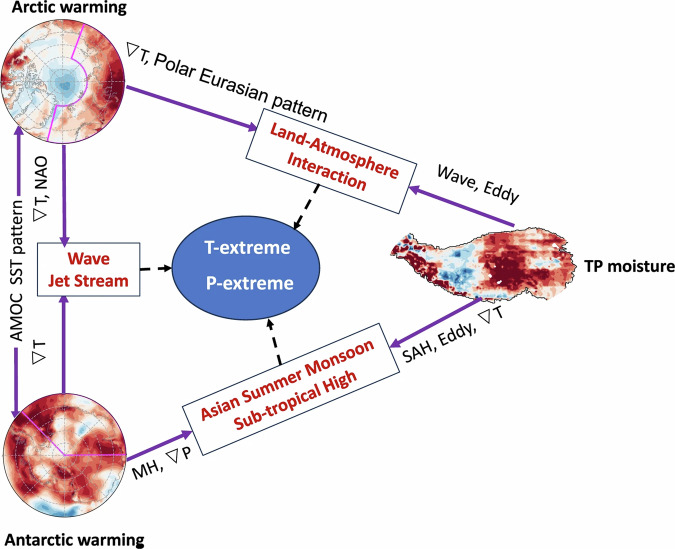
Schematic illustration of three Poles warming effects on the concurrent extremes in 2022. ∇T and ∇P denote temperature gradient and pressure gradient. MH and SAH refer to the Mascarene High and South Asia High, respectively. Temperature and precipitate extremes are referred to as T-extreme and P-extreme, respectively. The melting of ice and snow in the three Poles regions reduces surface albedo and increases surface absorption of shortwave radiation. This figure is from Zhang et al. (2024), and as part of that article is licensed under a Creative Commons Attribution 4.0 International License: http://creativecommons.org/licenses/by/4.0/. From: https://www.nature.com/articles/s41612-023-00553-6.

## Uncertainties in the major pathways

Existing studies attributing extreme events to sea ice and snow change are somewhat fragmented. Most of the studies employed observational analysis, atmosphere-only climate model experiments or a combination of them. However, uncertainties in these studies are still substantial. Based on these, we have synthesized a list of major uncertainties in the proposed pathways for sea ice and snow change to drive extreme events.

### Causality, nonlinearity and complexity of the derived relationships

**Fig. 8:**
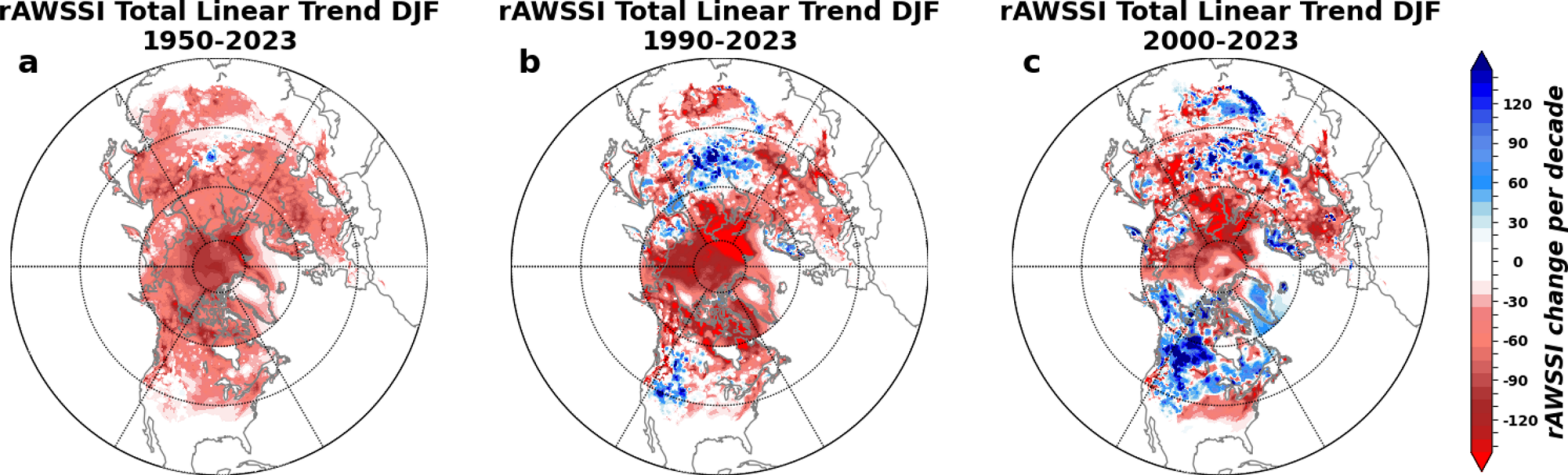
Severe winter weather decreased since 1950 but increased regionally since 1990 in Asia and since 2000 in North America. Decadal trends in the reanalysis-based Accumulated Winter Season Severity Index (rAWSSI) during three different time intervals: winters of (**a**) 1950/51–2022/23, (**b**) 1990/91–22/2023, and (**c**) 2000/01–2022/23. This figure is from Cohen et al. (2024), and as part of that article is licensed under a Creative Commons Attribution-NonCommercial-NoDerivatives 4.0 International License: http://creativecommons.org/licenses/by-nc-nd/4.0/. From: https://www.nature.com/articles/s43247-024-01720-0.

Observational analyses usually suffer from unclear causality particularly for relatively weak boundary forcing and for short observational records. Climate modeling studies generally suggest that the sea ice and snow forcing of the atmospheric circulation is relatively weak compared to internal variability^15,16^. Extreme events are complicated and manifest as a product of multiple drivers. Therefore, causality should be thoroughly considered for attributing extreme events to sea ice and snow change. Severe winter weather has exhibited a hemisphere-wide decrease since 1950 but showed regional increases in recent decades (Fig. 8). These contrast the relatively stable trends in sea ice and snow in the NH, suggesting the derived relationships between them and extreme events could be far more complicated than perceived. It has been shown that changes in cold spells and heatwaves in response to projected Arctic sea ice loss are nonlinear^185^.

Though most observational studies demonstrate a linear atmospheric response to sea ice melt, there are numerous modeling studies that demonstrate the atmospheric response can be nonlinear^186^. An early modeling paper showed that the hemispheric atmospheric response is very different to regional sea ice losses than to pan-Arctic sea ice loss. Furthermore, the atmospheric response to pan-Arctic sea ice loss differs from the linear addition of the responses to regional sea ice losses^187^. In observations, the correlations between NH weather patterns and regional sea ice loss also vary substantially across different Arctic subregions^188^. Sea ice loss in the Beaufort Sea and the Laptev Sea has contrasting impacts on the extreme cold events in the TP^189^. A different study showed that the NSPV response to sea ice loss is regionally dependent^186^. Sea ice loss in the North Atlantic sector resulted in a weaker NSPV and cooler temperatures across the midlatitudes while sea ice loss can lead to a stronger NSPV and warmer or cooler temperatures dependent on the magnitude of the forcing^190^. A later study confirmed both findings, that the atmospheric response to sea ice loss can be diametrically opposite depending on where the sea ice loss occurs and that these differences were due to tropospheric feedback mechanisms^191^.

### Signal-to-noise ratio, internal variability and competing effects

**Fig. 9:**
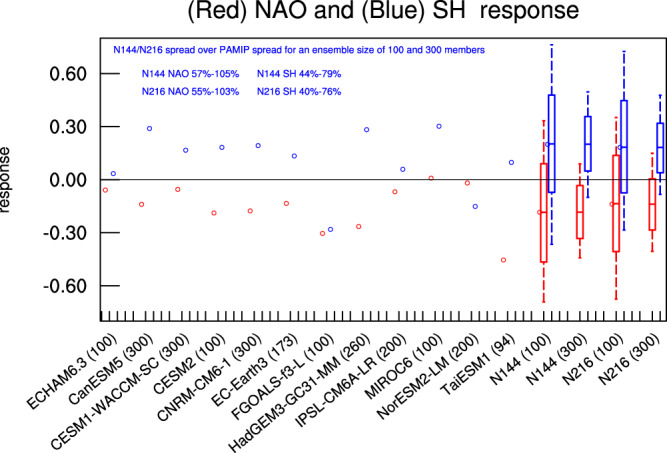
Internal atmospheric variability plays an important role in the inter-model differences in the PAMIP project. Normalized NAO (red circles) and Siberian High (SH; blue circles) responses computed for the PAMIP and those using the very large-ensemble climate simulations. Normalization is computed against the interannual variability of the NAO and SH in individual models. The ensemble size for each PAMIP model is given. Sub-sampled response of the (red) NAO and (blue) SH with 10,000 repetitions is displayed for both N144 and N216. Blue text at the top shows the ratio of sub-sampled spread to the inter-model spread in the PAMIP for an ensemble size of 100 and of 300, respectively, for upper and lower bound estimates. This figure is adapted from Ye et al. (2024), and as part of that article is licensed under a Creative Commons Attribution 4.0 International License: http://creativecommons.org/licenses/by/4.0/. From: https://www.nature.com/articles/s41612-023-00562-5.

Internal atmospheric and climate variability can obscure the detection of forced signals from sea ice and snow change^16,192^. This poses a major challenge in attributing extreme events to sea ice and snow change. Recent comprehensive modeling work suggests that the effect of Arctic sea ice loss is relatively weak compared to interannual variability of some internal atmospheric circulation mode, for example the North Atlantic Oscillation (NAO) and Siberian High^15,16^ (see also Fig. 9). The competing effects of thermodynamics and dynamics have led, for example, to a decrease in precipitation in Northern Eurasia^193^, contradicting the hypothetical increase in precipitation due to more moisture from a warming Arctic. The inter-model spread in the NAO response for the Polar Amplification Model Intercomparison Project (PAMIP) is largely explained by the internal atmospheric variability as obtained from the very large-ensemble climate simulations. Internal atmospheric variability is particularly an issue for both observation-based studies and small ensemble size climate simulations when studying the influence of Arctic sea ice loss^16^. It becomes even more challenging to detect the effect of Arctic sea ice loss on extreme events as it may require a much larger ensemble in modeling^16^. In addition, internal climate variability also needs to be addressed properly in future coupled climate modeling concerning the impacts of sea ice and snow change. Recent studies have also suggested that the impact of Arctic sea ice loss depends on and is modulated by some tropical modes of variability^194^. The tug of war between the Arctic and tropics in driving midlatitude response further complicates the detection of sea ice loss impacts^195,196^. The relative importance of sea ice and SST in driving climate and weather variability is also needed to be further studied^91^. For example, though Arctic sea ice loss is linked to the North Atlantic eddy-driven jet change in many studies, decadal variability of the North Atlantic eddy-driven jet could be closely linked to the North Atlantic SST^197^. For studies concerning snow change impacts, these issues have not been properly discussed and appreciated, though there have been some discussions on this issue^53,198,199^. Relatively small ensemble size climate simulations have been used so far concerning snow change impacts^31^. It is thus important that internal variability and competing effects are comprehensively addressed for attribution of extremes to sea ice and snow change.

### Sensitivity to metrics and methodologies

When quantifying AA and its impact on midlatitude climate and weather extremes, different metrics have been used in the literature^200^, which introduces uncertainties. For example, while most studies report that the Arctic has warmed 2–3 times faster than the global average, a recent study has reported a nearly fourfold warming^114^. One key issue when defining AA as a ratio of temperature changes is that the denominator may be close to zero during certain periods, which yields unrealistic ratios^201^. Different alternative metrics of AA have been proposed to alleviate this issue^202^. Moreover, different metrics for measuring e.g. the waviness in the jet and planetary waves can lead to conflicting results^116,117^. A possible explanation for these conflicting results is that AA leads to an increased spatial extent of jet meandering accompanied by reduced meridional amplitude; thus, different waviness metrics can give different results depending on which wave characteristic they focus on^203^.

In addition, different definitions of extreme events can lead to different results and conclusions^204,205^, which will complicate efforts to attribute their changes to a changing cryosphere. For example, the definition of heatwaves can be based on absolute thresholds^73^ or relative thresholds^27^. The lack of standardized definitions of extremes and disciplinary differences in defining and communicating them are a major barrier to achieve interdisciplinary synergy^204^.

For snow change impacts, there is a still a lack of a universal definition of snow droughts and consensus to best measure them^104^. This has also hindered the understanding of the impacts of snow droughts on warm seasons^104^.

### Experiment/model differences and model biases

**Fig. 10:**
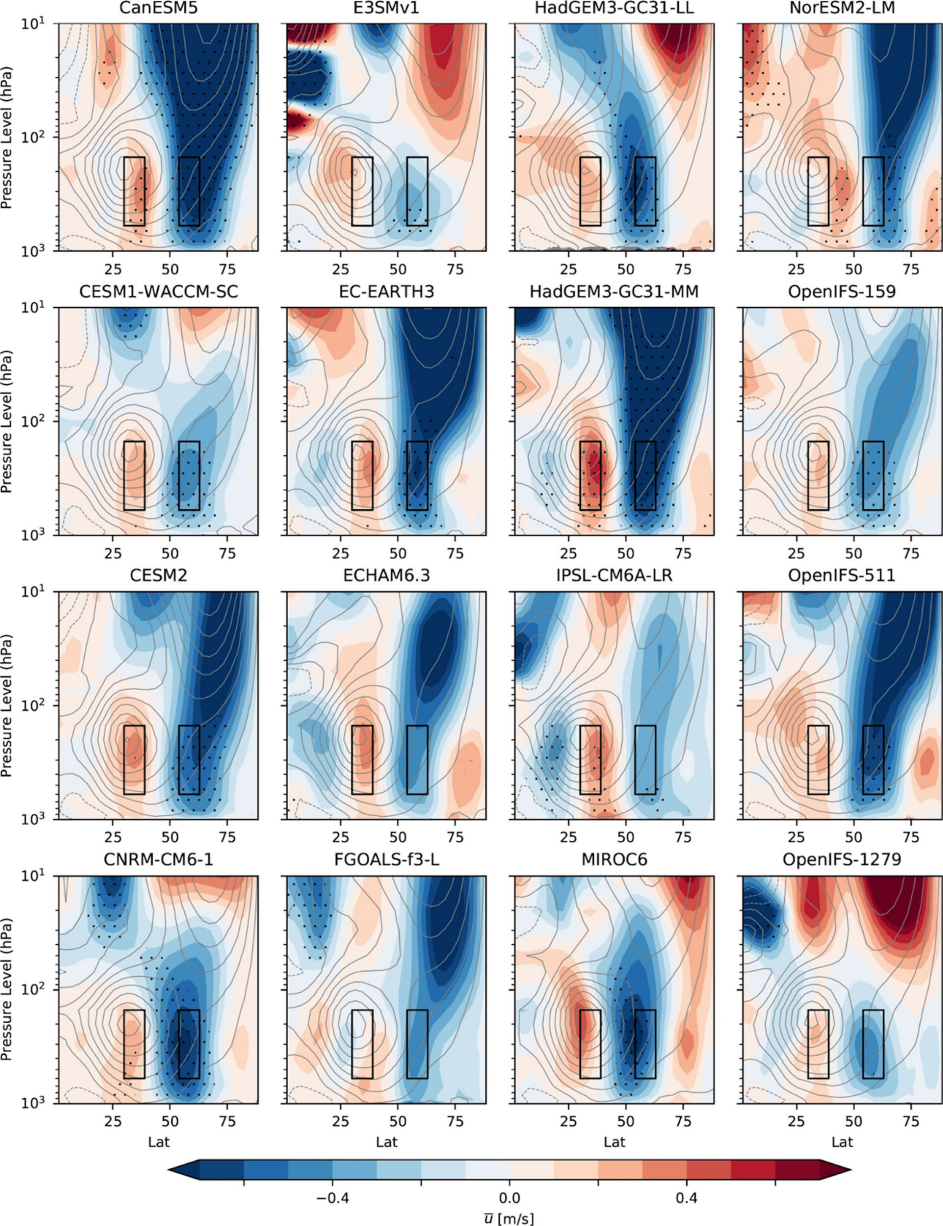
Relatively consistent tropospheric responses but contrasting stratospheric responses are found between different PAMIP models in response to projected Arctic sea ice loss. Zonally averaged DJF zonal wind response (ms^−1^) plotted as a function of latitude (°N) and height (pressure) for the ensemble mean of each of the models. The boxes show the regions used to compute the zonal wind response index. Stippling indicates where the ensemble mean response is significant (95% confidence interval). Contours show the climatological zonal mean winds (contour interval 5 ms^−1^ with negative contours dotted). This figure is taken from Smith et al. (2022), and as part of that article is licensed under a Creative Commons Attribution 4.0 International License: http://creativecommons.org/licenses/by/4.0/. From: https://www.nature.com/articles/s41467-022-28283-y.

Climate modeling has been a major tool in complementing observational analyses and determining causality in existing studies concerned with the impacts of sea ice and snow change on extreme events. Model-dependence of Arctic sea ice impacts and inconsistent modeling conclusions have been noted in existing studies^206,207^. In terms of studying sea ice impacts on climate and weather, the issues including model difference, experiment design, prescription of forcings and ocean–atmosphere coupling have been discussed in the PAMIP project and modeling efforts in PAMIP have addressed these issues in a comprehensive way^15,207^. Apart from internal variability, different model formulations such as model basic state^208^ may also contribute to the inter-model differences in the simulated impacts of Arctic sea ice loss^15,16^ (Figs. 9, 10). Coupled climate simulations are found to be essential for simulating remote effects of sea ice change and may also amplify the winter midlatitude wind response to Arctic sea ice loss^209^. For snow change, it has been suggested that imposing observed snow cover can help to capture the observed relationship between autumn Eurasian snow increase and winter atmospheric circulation^53^. Unrealistic representation of the unforced lower-stratospheric circulation in a model has affected how atmospheric circulation responds to autumn Siberian snow forcing^199^. The coupled climate models from the Coupled Model Intercomparison Project Phase 5 do not reproduce well the observed October Eurasian snow–Arctic Oscillation relationship, as the models poorly capture the downward propagation of stratospheric anomalies into the troposphere^210^. Nudging SWE towards observations has also been shown to improve the accuracy of modeled interannual surface air temperature variability^163^. The Land Surface, Snow and Soil moisture Model Intercomparison Project (LS3MIP)^211^ is a new opportunity to address these uncertainties in a comprehensive way. More results are expected to emerge from analysis of the LS3MIP project.

One major barrier to the development of model parameterizations specific to high-latitudes is the relatively short record and sparsity of observations, especially during the polar night. Processes on scales of 1–10 km and minutes to several hours are seldom resolved in the observational record, yet observational and modeling evidence indicates the importance of fine-scale features and temporal scales, especially in understanding Arctic interactions with the larger scales^212,213^. Atmospheric rivers transport a large fraction of the moisture into the Arctic^214–216^, yet there are contrasting views on their representativeness in the current conventional climate models^217,218^, and there are also substantial model differences in simulating/predicting them^219^.

In addition, global climate models exhibit large uncertainties and spread in simulating Arctic change and linkages with the midlatitudes^220,221^. The causes of this large inter-model spread in Arctic warming relate to many possible modeling deficiencies. In particular, uncertainties in model parameterizations handicap our ability to predict future Arctic SIE and its potential interaction with midlatitudes. Cloud microphysics, convection, boundary layer processes, and surface turbulent flux parameterizations are primarily developed to ensure accurate forecasts in the tropics and the midlatitudes may not be applicable at high-latitudes^222^. Furthermore, inaccuracies outside of the Arctic, such as tropical convective parameterizations, could contribute to the uncertainty in the large-scale circulation^223,224^ that contributes to Arctic warming. Deficiencies have also been identified in how models approximate the surface mass and energy budgets, including: surface albedo parameterizations^225^; sea ice rheology^226^; fluxes across the atmosphere-ice-ocean boundary layer^225,227^; and cloud radiative properties. This wide range of relevant processes requires accurate representation of Arctic sea ice^209^ which is currently challenging for climate models. Given these limitations, it is not surprising that the level of uncertainty about present and future Arctic warming and influence is considerable.

### Incomplete processes understanding of intermediate pathways

Some intermediate pathways such as troposphere–stratosphere coupling, ocean–atmosphere coupling and land surface process are still highly uncertain in bridging the impacts of sea ice and snow change on climate and weather extremes. There is still a need to advance understanding of stratospheric variability related to tropospheric forcing in order to better understand the impact of sea ice and snow change on stratospheric variability and climate/weather variability^31,228^. The stratospheric response to sea ice loss exhibits diverse magnitudes and even opposite signs between different models in the PAMIP project^15^ (see also Fig. 10). Land surface processes, mainly the snow hydrological effect, have long been proposed and studied. However, the snow hydrological effect is still poorly understood and there is no consensus on its importance in terms of linking snow change to climate and weather variability^129,161,162,229^. One debated issue is the persistence of soil moisture memory following snowmelt water infiltration^162,229,230^. This process also demands a comprehensive understanding of soil moisture–atmosphere coupling^166,231^, which further adds to the dimension of complexity of snow hydrological effect. A recent single-model SWE pacemaker experiment of land–atmosphere coupling for the period 1901–2010 has suggested that all these land surface processes are important for snow change to significantly impact atmospheric air temperature variability^163^. In terms of the oceanic pathway, studies are still relatively limited in terms of ocean–atmosphere interactions in bridging the influence of sea ice and snow change^60,173–176^. On the other hand, the record low Arctic SIE in 2012 was found to be driven mainly by the back-to-back La Niña events during 2010–2011^232^. Much is therefore still unknown for the bridging role of ocean–atmosphere coupling in linking sea ice and snow change with extreme events.

### Timescale-dependence/intermittency

Atmospheric blocking and stratosphere–troposphere coupling are both crucial for Arctic/midlatitude weather linkages, yet both blocking and stratospheric influence are intermittent and have preferred geographic locations and sub-seasonal duration (~10–20 days)^143^. Attributing the cause of any particular extreme event or series of related events to one or more of the many forcings or influences that can excite them, including natural variability, is one of the most challenging aspects of understanding the consequences of climate change including changes to the cryosphere. In any given season, year, or decade a different combination of factors dominates with alternating levels of influence, magnitudes of response, timing and location of extremes^143^. Observational analysis alone cannot determine causal relationships, while model sensitivity studies based on numerical models are deficient in fully simulating the multiple interacting factors that cause extreme weather events and the correct response for both timing and magnitude^85,207^, as discussed above. Assessment of relative and intermittent contributions by internal atmospheric chaotic variability, identification of cause and effect, and the lack of consensus between model and observational studies^65,85,119^ complicate the problem.

## Detecting and attributing cryospheric driving of climate and weather extremes – emerging opportunities and suggested ways forward

Further advances in attribution of climate and weather extremes to sea ice and snow have considerable scientific and societal benefits, including improving predictions of extreme events and contributing to adaptation to climate change. However, this has so far been hindered by fragmented studies and uncertainties in physical pathways. In view of the existing challenges and climate change, we propose some ways forward to facilitate community efforts in addressing the limitations of our existing work and exploring emerging methodologies and opportunities to advance the area of research.

### Improving observational networks and process-based understanding

Owing to the extreme environmental conditions and complex surface conditions, observational data for both sea ice and snow are still relatively limited. High-quality, long-term observations of sea ice and snow are critical for studying their interactions with the climate system, particularly in terms of ice–ocean–atmosphere and snow–land surface–atmosphere interactions. Thus, integrating existing networks of observations and expanding observational capability will be important for such purposes. Process-level observations and modeling are particularly important for studying the local and regional atmospheric responses to sea ice and snow change. For example, marine cold air outbreaks (MCAOs) are important for the formation of marine cyclones and polar lows, which cause severe weather conditions at high latitudes^233^. It is suggested that retreat of sea-ice will increase the strength of MCAOs in regions of recently exposed ocean over the marginal ice zone in the Nordic seas^234^ and future sea-ice reduction may broaden the area of MCAOs^234^. Ground/ship/ice-based remote sensing of the Arctic atmosphere should be further expanded to better characterize the vertical profiles throughout the Arctic troposphere. Dedicated field campaigns, by regular observations at well-instrumented super sites (e.g., the International Arctic Systems for Observing the Atmosphere and the National Aeronautics and Space Administration’s SnowEx campaign^235^), and by satellites will provide valuable observations to complement process understanding. Better monitoring and observations of snow change in high mountains^236^ is also important. Climate modeling and observational analysis will need to obtain a better understanding of the forced versus internal variability of the cryosphere, for example the sea ice loss in the recent decades^237^. This will benefit the process understanding of sea ice and snow influencing extreme events by separating climate change signals from internal climate variability. It will also contribute to the attribution of extreme events in terms of human-driven versus naturally-driven causes. In addition, enhanced sea surface/land surface observations and data integration are also important. Improving observational infrastructures and systems via international programs and campaigns will be needed to achieve these goals.

### Addressing causality, nonlinearity and complexity

Future studies will need to address the causality and complexity of sea ice and snow driving extremes by combining multiple lines of investigation and evidence, particularly in terms of quantitative evidence. This will include using available new long-term observational and reanalysis data, thorough statistical testing of obtained relationships, and combining observational analysis with targeted model experiments or analyzing existing model output. A hierarchy of modeling, from idealized linear models to fully coupled Earth system models, will be useful to understand the complicated processes involved in linking sea ice and snow change to extreme events. Paleoclimate data and analysis is another valuable line of evidence that can be added to the understanding and attribution. In particular, considering that there are competing factors for extreme events and internal variability that are relatively large, it is important to address causality and complexity from the perspective of multifaceted evidence. Recent success in weather forecast-based attribution of extreme events^238^, assimilating land surface data in coupled simulations^80^ and using causal networks for evaluating climate model simulations and constraining projections^239–241^ can be considered as new ways for attributing extremes to sea ice and snow change. This will provide additional evidence to support and complement existing analyses and model experiments. In addition, improving understanding of ocean–sea ice–atmosphere interactions and snow–atmosphere interactions and their representation in climate models is an important step towards improving climate models and reducing model biases. This will further strengthen the credibility of modeling evidence derived from model experiments when attributing extremes to sea ice and snow change.

### Large-ensemble approaches and the application of artificial intelligence

Large-ensemble approaches have many advantages in helping with detection of forced response to sea ice and snow change. Future studies will need to consider large-ensemble coupled/uncoupled climate simulations in single- or multi-model settings in attributing extremes to sea ice and snow change so that robust sampling and quantification of internal variability can help with the detection and attribution. Large-ensemble simulations will also provide opportunities for further studying flow/background-dependence and preconditioning of the impacts of sea ice and snow change. Artificial intelligence (AI) such as machine learning has been applied to tackle many problems^242–244^ and has the potential to aid the attribution of extremes to sea ice and snow change^245^. Applying AI to the wealth of climate model output and future model simulations output will potentially unravel new linkages between sea ice, snow and extreme events. The specific areas AI will prove particularly useful are detection of linear/nonlinear links between sea ice, snow and extremes; prediction of future extremes that are linked with sea ice and snow change; and improving observations, for example extending past observational records, filling gaps and bias-correcting in observations.

### Achieving synergy of methodologies and disciplines

Different metrics and methodologies have been used to study the impacts of sea ice and snow change on extreme events. We suggest future research tap into the diverse methodologies currently in use and for future exploration to achieve a synergy of methods. In particular, a standardization of metrics that can achieve synergy of different metrics and methodologies will bring further advances in the research. Further studies into the combined influence of sea ice and snow change on extreme events are also important for highlighting the climatic feedback of a changing cryosphere. Further, enhancing and promoting interdisciplinary collaboration in attributing extremes to sea ice and snow change and in assessing their impacts is also an important step towards improving scientific rigours and advances in the area of research. This will help advance the understanding of cascading impacts of a changing cryosphere on the climate system and the environment.

### Widening context and international collaboration

Sea ice and snow are undoubtedly only two of the many factors that drive extreme events in the coupled climate system. For example, SST anomaly, more-persistent double jets, nonlinear interactions between slow- and fast-moving components of the atmospheric circulation along with low soil moisture have been suggested to drive some extreme events^246–249^. In addition, sea ice and snow can be merely an intermediate state which bridges the remote drivers of climate and weather extremes in some cases. Therefore, it is useful to contextualize attribution of extremes to sea ice and snow for a wider context in a changing climate, so that how and to what extent sea ice and snow play a role in driving extreme events are better understood and quantified. This will help to better guide future research and attribution studies in terms of incorporating cryospheric elements such as sea ice and snow into extreme events research, attribution and prediction. For example, combining both El Niño signal and the TP snow cover in a linear regression model predicts well the extreme winter conditions of 2009/10 in the NH^250^. Finally, international programs and modeling consortia will be vital for advancing research and attribution of extremes to sea ice and snow change. For example, the PAMIP project has been a success in advancing the study of sea ice loss impacts^207^. However, there is still a lack of international collaborations and efforts in attribution of their impacts on extreme events. Embedding this direction in existing international programs or in future international programs will provide promising prospects in advancing the attribution of extremes to sea ice and snow change.

Polar regions and high latitudes are sensitive and vulnerable to climate change. Changes in extremes in the polar regions for example, the documented string of Antarctic extreme events^251^, and the occurrence of tipping points in the climate system including the loss of summer Arctic sea ice^252^ would have far-reaching implications. In particular, an emerging new Arctic has been proposed where extremes are the norm^253^. The rapid loss of multiyear sea ice in the Arctic during 2005/2007^254,255^ may have changed the ice dynamics that could affect transpolar drift and mean ice thickness, leading to a state change. A state change in the polar and high-latitude climate system would potentially transform how we study and assess the cryospheric impacts on climate and weather extremes. Examples would include the loss of continuity and the loss of smooth trends in the time series of cryospheric observations, which may not be well represented in available datasets that we rely on. Therefore, future research on tipping points and systemic transitions in the cryospheric system is highly relevant for understanding the changing cryosphere and its impacts on climate and weather extremes.

## Data Availability

Data that support the analysis in this study are available from National Snow and Ice Data Center, Rutgers University Global Snow Lab, GlobSnow Data (https://www.globsnow.info/), Met Office Hadley Centre observations datasets, and MODIS (https://modis.gsfc.nasa.gov/).
